# The Effectiveness of Acupuncture on Headache Intensity and Frequency in Patients With Tension-Type Headache: A Systematic Review and Meta-Analysis

**DOI:** 10.7759/cureus.14237

**Published:** 2021-04-01

**Authors:** Spyridon Kolokotsios, Alexandra Stamouli, Ioannis Koukoulithras, Minas Plexousakis, Gianna Drousia

**Affiliations:** 1 Department of Physical Therapy, University Hospital, University of West Attica, Athens, GRC; 2 Department of Orthopaedic Surgery, Faculty of Medicine, University of Ioannina, Athens, GRC

**Keywords:** acupuncture, systematic review and meta-analysis, headache, tension type headache

## Abstract

Introduction

Headache disorders are one of the most common health problems. Tension-type headache (TTH) is the most prevalent type of primary headache in adults. Several conservative treatments have been used for the management of TTH, such as analgesics, acupuncture, manual therapy, spinal mobilization. This study aims to examine the effectiveness of acupuncture in patients with TTH.

Methods and materials

PubMed, PEDro database, Cochrane Library, and Google Scholar were searched from January 2000 until February 2021, as well as the reference lists from identified articles. Studies of various acupuncture types were included, but only randomized controlled trials and clinical trials were selected. The studies were screened using the Preferred Reporting Items for Systematic Reviews and Meta-Analyses (PRISMA) question. Details about the type of acupuncture, sample size, outcome measures, results, and statistical significance were extracted from the selected studies. A short-term (after the last treatment) and long-term meta-analysis for pain intensity and frequency of headaches was conducted. The I^2^ index, as well as the x^2^ test, were used to determine the heterogeneity between studies. A random-effects meta-analysis was carried out.

Results

From all the studies found in the mentioned databases, only 15 studies with 1272 participants met the criteria. In the meta-analysis, four studies with 557 participants were included. The headaches' frequency after the last treatment was not significantly lower in the acupuncture group than in the placebo/sham group (mean difference: -1.53 [CI: -4.73, 1.67]). However, acupuncture seems to improve the frequency of headaches in the long term, although the results were not statistically significant p=0.06. Furthermore, there was a reduction of 1.55 days per month of headaches in the acupuncture group versus placebo (mean difference: -1.55 [CI: -3.19, 0.09]), but it was not statistically significant. The visual analog scale (VAS) score of the acupuncture group slightly reduced (-0.29) compared with the control group after the last treatment (mean difference: -0.29 [CI: -1.21, 0.62]), although the two groups were not statistically significant p=0.53. In the long term, acupuncture demonstrated a statistical (p=0.009) and clinical benefit compared with placebo/sham. Statistical analyses between the two groups showed a reduction of 0.41 in the VAS scale at the acupuncture group (mean difference: -0.41 [CI: -0.72, -0.10]).

Conclusion

Overall, after the meta-analysis of articles with high methodological quality, acupuncture's effectiveness compared to sham seems to be statistically non-significant on headache intensity and frequency in patients with TTH after the treatment. Both headache intensity and frequency were reduced in the long term, although only in the pain intensity, the results were statistically significant. Therefore, more studies on this topic should be conducted to examine its effectiveness in headache frequency and intensity.

## Introduction

At present, headache disorders (HD) are one of the most common health problems, causing substantial pain and disability in the head, while the pain can also be spread and radiated to the neck [1,2]. Headaches are classified as primary or secondary, based on the clinical presentation. Primary headaches result from dysfunction or overactivity of the heads’ structures, without a clear underlying cause. On the other hand, secondary HD are conditions such as neck trauma, vascular disorders, musculoskeletal problems, and arthritic changes [1]. Tension-type headache (TTH) is the most prevalent type of primary HD in adults, associated with a high economic burden for society, while patients’ moods and general life activity are affected [1].

According to previous research, TTH pathogenesis remains unclear, although the etiological factors are possibly related to peripheral mechanisms (myofascial nociception) and central mechanisms (sensitization and inadequate endogenous pain control) [3]. The development of TTH is probably associated with myofascial trigger points (TrPs). The TrPs in the head and neck muscles are painful on compression, and they are related to refer to pain - tenderness, motor dysfunction, and autonomic phenomena [4]. Increased muscle stiffness in the trapezius muscle and rectus capitis posterior minor (suboccipital muscles) in TTH patients has been demonstrated. The muscle stiffness does not differ between headache outbreaks and non-headache days [4].

Several conservative treatments have been proposed for the management of HD. The most common treatments include medication, physical therapy, and relaxation/cognitive therapy [1,5]. Various physical therapy interventions have been suggested to treat headaches. For example, manual therapy, massage, spinal mobilization or manipulation, soft tissue release, and acupuncture are widely utilized [1]. However, studies revealed that alternative medicine therapies, such as acupuncture, are the most used treatments requested by patients with headaches [5].

Acupuncture is defined as the needling of specific points of the body. It was initially developed as a part of Chinese medicine, wherein the purpose of treatment was to release the flow of the body’s vital energy by stimulating points along energy pathways. However, acupuncture practitioners have abolished these concepts and approach medical acupuncture concerning neurophysiology [2].

There is a widely held belief, without proof, that using acupuncture as an intervention to treat TrPs in the muscles of the head and neck may reduce the frequency and intensity of headaches [1,3,6]. Notwithstanding the growing use of acupuncture in treating headaches, its clinical effectiveness is still debatable due to a lack of proper reviews [1]. So, it stands to reason that this meta-analysis aims to examine acupuncture's effectiveness in TTH patients. The aim of this study is to examine the effectiveness of acupuncture in comparison with sham or in a combination with interventions on headache frequency and pain intensity in patients with tension-type headaches.

## Materials and methods

This study was conducted following the preferred reporting items for systematic reviews and meta-analyses guidelines (PRISMA). The great majority of the studies found during our initial search focused on dry needling rather than acupuncture. Linde et al. [7] conducted a detailed study on this subject, but they included only randomized controlled trials (RCTs). Therefore this systematic review and meta-analysis aimed to investigate both RCTs and non-randomized clinical trials as well as add the newest data regarding TTH and its management with the use of acupuncture.

Search strategy

The search was conducted through four electronic databases: MEDLINE via PubMed, CENTRAL via Cochrane Library, PEDro, and Google Scholar. Furthermore, the reference list of the articles found was examined to discover more studies on the same topic. Only articles between January 2000 and February 2021 were included. The fact that research on this subject has never been conducted before was the purpose that we choose to examine this 21 years. Also, both RCTs and clinical trials were included because of the lack of bibliography in articles about the management of tension-type headache using acupuncture. The search strategy was based on an algorithm made with the use of MeSH terms (Medical Subject Headings): “acupuncture” OR “acupuncture therapy” AND “tension-type headache.”

Study selection

The Population Intervention Comparison Outcome question (PICO) was used to select studies (Table 1). Three authors carried out the primary screening of the articles found in the four electronic databases and selected articles based on the title and abstract. Each one of them searched all databases independently, and then the outcomes were compared. In the case of different outcomes and disagreement between them, the issues were resolved with the two other authors' intervention. The full text of screened articles was studied if one of the treatment arms of RCTs or clinical trials was about tension-type headaches and the efficacy of acupuncture as a treatment. Although RCTs are considered for ranking second after systematic reviews - which include only RCTs regarding internal validity - and their design is more suitable for research, in this study we included RCTs and clinical trials because of the limited bibliography found [8].

**Table 1:** PICO Question

P	Population/Problem/Patient	Patients with tension-type headache
I	Intervention	Acupuncture
C	Comparison	Physical and occupational therapy
O	Outcome of interest	Reduction of headache frequency and intensity

Exclusion criteria

Specific exclusion criteria were established from the beginning of the study. Trials published before 2000 were excluded, and those written in different languages other than English or Greek. Also, articles that could not be found in the full text were not included in the research. 

Evaluation of methodological quality

The assessment of the methodological quality of included articles was performed by two authors independently and was based on the criteria of the Physiotherapy Evidence Database (PEDro) scale. The PEDro scale has 11 criteria, 10 of which evaluate the study's internal validity (criteria 2-9) and statistical information sufficiency (criteria 10, 11) [9]. Methodological quality was categorized as «low» for articles with a score of less than 3/10, «moderate» for articles with a score of 4-6/10, and «high» for articles with a score of 7-10/10. Differences between authors regarding the quality appraisal and extracted data were resolved through discussion with the other authors and team members.

Data extraction

Data were extracted from the selected studies by three authors regarding interventions, sample size, duration, follow-up, outcome measures, outcomes, and statistical significance independently. Especially, the primary outcomes that were extracted were the headache intensity evaluated with the VAS score and frequency (days/month).

Assessment of risk of bias

To evaluate the risk of bias, the Cochrane reviews approach was used to determine the accuracy of the included studies' results. The assessment followed the criteria of the Grading of Recommendations Assessment, Development and Evaluation (GRADE) [10]: allocation concealment, adequate sequence generation (selection bias), blinding of participants and personnel (performance bias), incomplete outcome data addressed (attrition bias), free of selective reporting and free of other bias. Two of the authors graded the included articles, and if disagreement occurred, a fourth author would resolve the issue.

Meta-analysis

A meta-analysis was conducted to determine the effectiveness of acupuncture as monotherapy versus sham/placebo in patients with tension-type headaches. The frequency of headaches (days per month) and the pain intensity (VAS score 0-10) were the primary outcomes. For the trials that a meta-analysis was not possible (not relevant for the meta-analysis topic, not sufficient data), the results were presented as a narrative synthesis. Statistical analysis for the pain intensity and frequency of headaches were performed using the Review Manager 5.4 software (Cochrane Collaboration, Copenhagen). The odds ratio (OR) was calculated for continuous data, and the level of statistical significance was set at p<0.05. Analyses for the clinical and RCTs were conducted using a random-effects model of the Mantel-Haenszel method. The overall effects and the heterogeneity of continuous data across trials were examined by calculating Z values and χ^2^ distributed Cochrane Q values, respectively. The I^2^-test of heterogeneity was also conducted to obtain I^2 ^values based on the formula (I^2^ = 100%⁎ (Q − degree of freedom)/Q)) (Higgins et al. [11]). We considered I^2^ less than 25% to indicate low heterogeneity and I^2^ greater than 75% to indicate considerable heterogeneity.

## Results

Literature review results

**Figure 1:**
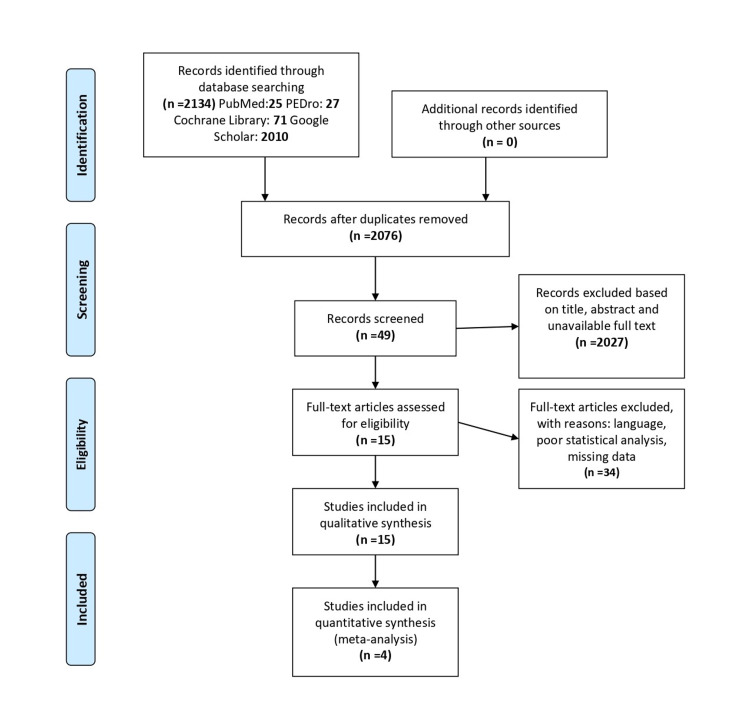
Preferred Reporting Items for Systematic Reviews and Meta-Analyses (PRISMA) flow diagram

The systematic review and meta-analysis process is depicted in the flow diagram displayed in (Figure 1). From the initial database search, 2134 articles were collected from four electronic databases (PubMed: 25; PEDro: 27; Cochrane Library: 71; Google scholar: 2010;). Studies were reduced to 15 following the removal of duplicates and applying the established exclusion criteria after their assessment. Based on the inclusion, exclusion criteria, and the design exhibited for the present systematic review, 15 articles (RCTs and clinical trials) were included for qualitative synthesis, and 1267 patients were examined. Subsequently, RCTs and clinical trials were identified and excluded with reasons for the scope of meta-analysis. The information of the selected articles displayed in Table 2. Ultimately, of the remaining 15 articles, 11 did not meet the inclusion criteria for the meta-analysis. A meta-analysis for pain intensity and frequency of headaches was conducted after the end of treatment and after the last follow-up (long term). For the meta-analysis of headache frequency, as well as for the meta-analysis of headache intensity, we used four studies. Although, in the second meta-analysis for the pain intensity after the last treatment, only three studies were eligible due to the absence of data.

**Table 2:** Characteristics of articles Visual Analogue Scale (VAS), Clinical Global Impressions (CGI), Mechanical Pressure Pain Threshold (PPT), General Health Questionnaire (GHQ), Depression Score (D-S), Freiburg Questionnaire of Coping with Illness (FQCI), Nottingham Health Profile (NHP), Everyday Life Questionnaire (ELQ), Life Quality Scale (LS), Range of Motion (ROM), 12-Item Short-Form Health Survey (SF-12), Minor Symptom Evaluation Profile questionnaire (MSEP), Dry Needling (DN), Sham Dry Needling (SDN)

	Authors	Intervention	Sample size	Duration	Follow up	Outcome measures	Outcomes	Statistical significance (CI/p)
1	Karst et al. (2000) [12]	Acupuncture vs Placebo	N=39 Acupuncture n= 21 Placebo n=18	2 treatments per week and ten treatments in total	Follow-up 1: last day of treatment; follow-up 2: 6 weeks after treatment.	VAS CGI Frequency per month PPT	Six weeks after the end of treatment no significant differences between placebo and verum could be observed with respect to visual analogue scale and frequency of headache attacks.	VAS scores significantly decreased but without difference between placebo and verum group P=0.001
2	White et al. (2000) [13]	Acupuncture vs Placebo	N=44 Acupuncture n= 22 Placebo n=22	1 treatment/week for 6 weeks	The data were analysed in four periods of 3 weeks (run-in, last 3 weeks of treatment, early follow up, late follow up).	VAS GHQ Headache duration Mean General Health Questionnaire mean score Social functioning disruption scale	No significant differences were found between the changes in the two groups for any measure at any time point.	P>0.05
3	Karst et al. (2001) [14]	Acupuncture vs Placebo	N=69 Acupuncture n= 34 Placebo n=35	2 treatments/week for a total of 10 treatments	Follow-up 1, last day of treatment; follow-up 2, 6 weeks after treatment; follow-up 3, 5 months after treatment	VAS Frequency per month (days), Analgesics per month D-S FQCI 1 FQCI 5 CGI NHP ELQ LS	No significant differences between placebo and verum with respect to visual analogue scale and frequency of headache attacks could be observed immediately, 6 weeks, and 5 months after the end of treatment. There was a significant but weak improvement in quality of life parameters (clinical global impressions, Nottingham Health Profile) after verum treatment.	
4	Karakurum et al. (2001) [19]	Dry needling vs Placebo	N=30 Dry needling n=15 placebo n=15	4 weeks	After the last treatment (week 4)	Tenderness scores ROM	Mean headache indices improved significantly after treatment, both in the treatment group and in the placebo group, but the difference between the two groups was insignificant. In the treatment group, the tenderness score and the neck ROM limitation score were significantly improved after treatment, while there was no significant improvement in the placebo group.	P<0.05
5	Xue et al. (2004) [22]	Electroacupuncture (Group A) vs Sham Electroacupuncture (Group B)	N=40 Group A n=20 Group B n=20	Phase 1 (4 weeks) Phase 2 ( 4 weeks )	(3 months after phase 2)	VAS PPT Headache disability and sickness impact	There were no significant differences between the 2 groups at baseline and at the end of follow up	P<0.05
6	Ebneshahidi et al. (2005) [25]	Laser acupuncture (Group A) vs sham laser acupuncture (Group B)	N=50 Group A n=25 Group B n=25	Three times per week for 10 sessions	Three assessments were made at monthly intervals after the last session.	VAS Duration of attacks Days/month with a headache	There were significant differences between groups in changes from baseline in months one, two, and three, in median score for headache intensity, median duration of attacks, and median number of days with headache per month.	P<0.001
7	Söderberg et al. (2006) [16]	Acupuncture (ACU) vs Physical training (PT) vs Relaxation training (RT)	N=90 Acupuncture n= 30 Physical training n = 30 Relaxation training n = 30	2.5–3 months	The first follow-up was directly after treatment with collected diaries. The next follow-up was at the 3rd month and the last follow-up was at the 6th.	VAS Headache-free days and Headache-free periods.	There were no significant differences in headache intensity, headache-free days, or headache-free periods between the 3 treatment groups during baseline ratings 4 weeks prior to the treatment period. The relaxation group reported a significantly higher number of headache-free periods and a significantly higher number of headache-free days compared with the acupuncture group immediately after the last treatment. There were no other significant group differences between the study groups at any time point.	Relaxation training vs ACU group: The RT group reported a significantly higher number of headache-free periods (P< 0.05) and a significantly higher number of headache-free days (P < 0.01) compared with the ACU group immediately after the last treatment.
8	Endres et al. (2007) [15]	Acupuncture (verum points) vs Sham acupuncture	N=409 Verum n= 209, Sham n= 200	Ten 30-min sessions were given over a six-week period	First, follow up directly after treatment. The next follow up was at 3 months and the last follow up was at the 6 months	Daily diaries of headaches and medication use every day, Von Korff chronic pain grade scale in a modified 3-month version the German version of the SF-12 (12-Item Short-Form Health Survey) for health-related quality of life	>50% reduction in headache days/month at six months and no use of excluded concomitant medication or other therapies	Verum was superior to sham for most secondary endpoints, including headache days (1.8 fewer; 95% CI 0.6, 3.0; p=0.004) and the International Headache Society response criterion (66% vs. 55% response, risk difference 12%, 95% CI: 2%–21%; p=0.024)
9	Wang et al. (2007) [24]	Acupuncture like electrical stimulator vs sham stimulator	N=36 Treatment group n= 18 Placebo group n=18	4 weeks	2 weeks, 4 weeks, 6 weeks	Duration of headache pain was calculated as hours with a headache each day. Pain intensity was scored on a 0 to 10 cm visual analogue scale. draw the area of pain and describe the quality of pain on a Danish version of the McGill Pain questionnaire at each stage. The pain rating indices of the sensory, affective, evaluative, and miscellaneous dimensions of pain were calculated in accordance with Melzack. The frequency of headache attack and medicine consumption was recorded	The pain duration was shortened at Treat-1 and pain intensity was decreased at Treat-1 and Treat-2 compared with baseline. The overall evaluation of the 2 treatments indicated improvements in both the treatment and the placebo groups, but with no significant difference between the groups	Improvements in both the treatment and the placebo groups, but with no significant difference between the groups (P>0.061).
10	Söderberg et al. (2011) [17]	Acupuncture vs Relaxation training vs Physical training	N=90 Acupuncture n=30 Relaxation training n=30 Physical training n=30	2,5-3 months	3 and 6 months after the end of treatment	Minor Symptom Evaluation Profile questionnaire (MSEP) VAS	There were no significant differences in TTH and quality of life between the 3 groups directly after treatment. Although at the 3-month follow-up, the physical training group showed the strongest improvement in TTH compared to acupuncture. Though at the 6-month follow up no difference between the 3 groups was detected but only a slight increase in the relaxation training group.	After treatment: no statistical significance found 3-month follow up: ACU vs physical training group (p=0.036) 6-month follow up: no significant differences
11	Chassot et al. (2015) [23]	Electroacupuncture vs Sham electroacupuncture	N=34 Electroacupuncture n=18 Sham n=16	2 times/week for 5 weeks and again after 2 weeks the same pattern with the other technique	No, follow up after the end of treatment	Hamilton Depression Rating Scale Pain Catastrophizing Scale score Short-Form Headache Impact Test (HIT-6) VAS	EA was found to have a statistically significant result in alleviating pain after the treatment compared to sham but no long-last effect was detected.	EA vs Sham: p=0.005 VAS score ( 2.38 ± 1.77 and 3.02 ± 2.49)
12	Georgoudis et al. (2018) [26]	Acupuncture/stretching vs Acupuncture/stretching plus Physiotherapy techniques(microwave diathermy and myofascial release)	N=44 Acupuncture/stretching n=20 Acupuncture/stretching plus Physiotherapy techniques n=24	4 weeks	No follow up after the end of treatment	PPT VAS	In both groups, the provided interventions were found to have a statistically significant result while further improvement was detected in the group with the additional physiotherapy techniques.	An improvement was noted in both groups/treatments regarding the main outcome measure PPT, all the way from the first to fifth and the 10th treatment, at all measuring sites and at all measurements in both groups (p< .001). When comparing the 2 groups, differences were noted after the 10th treatment (p< .05).
13	Gildir et al. (2019) [20]	Dry needling vs Sham dry needling	N=161 Dry needling n=80 Sham dry needling n=81	3 times/week for 2 weeks	1 month after the end of treatment	Short Form-36 VAS	There was found a statistically significant difference regarding the intensity, frequency, and duration of headache and the quality of life in both groups after treatment as well as at the follow-up. The only exception was the headache duration in the period from post-treatment to follow-up that wasn't found important.	DN: p=0.001(intensity, frequency, duration, quality of life) SDN:p=0.003 (intensity), p=0.001 (frequency, duration, quality of life)
14	Kamali et al. (2019) [21]	Dry needling vs Friction massage	N=40 Dry needling n=20 Friction massage n=20	3 times/week for 1 week	No follow-up after the end of treatment	Algometer Goniometer VAS	Both interventions improved the frequency, intensity of headache as well as pain threshold at the trigger points through dry needling was found to have a more statistically significant result than friction massage. However, neither of the two methods had any effect on cervical range of motion except for extension, which increased in the dry needling group.	DN: headache intensity, frequency, pain threshold, cervical range of motion (extension) p<0.005 Friction massage: headache intensity, frequency, pain threshold p<0.005 DN vs Friction massage(pain threshold):p=0.008
15	Schiller et al. (2021) [18]	Acupuncture vs Medical training therapy vs Usual care vs Acupuncture plus medical training	N=96 Usual care n=24 Acupuncture n=24 Medical training n=24 Acupuncture plus medical training n=24	2 times/week for 6 weeks	3 and 6 months after the end of treatment	Pain intensity Frequency of headache Duration of headache Use of headache medication	The combination of acupuncture and medical training therapy significantly reduced mean pain intensity compared to usual care(p<0.012). In contrast, neither acupuncture nor medical training therapy differed significantly from usual care. No between-group differences were found in headache frequency, mean duration of headache episodes, and pain medication intake. At 3 months, the majority of all patients showed a reduction of at least 50% in headache frequency. At 6 months, significantly higher responder rates were found in all intervention groups compared to usual care.	Acupuncture plus medical training therapy vs usual care: mean pain intensity p=0.012 Acupuncture or medical training therapy vs usual care: no statistical significance 3-month and 6-month follow up: headache frequency reduced but not statistically significant.

Methodological quality of selected articles

Specifically, 15 studies were chosen based on our inclusion criteria (Table 2). Out of these studies, seven used acupuncture as the main intervention and were evaluated as high methodological quality (Karst et al. [12], White et al. [13], Karst et al. [14], Endres et al. [15], Söderberg et al. [16], Söderberg et al. [17], Schiller et al. [18]). Some compared acupuncture to sham while others with other techniques. Three studies utilized dry needling, and they were all evaluated as moderate methodological quality (Karakurum et al. [19], Gildir et al. [20], Kamali et al. [21]). Moreover, three studies researched the effects of electroacupuncture, and all of them were evaluated as high quality (Xue et al. [22], Chassot et al. [23]), with one of them scoring 10/10 on the PEDro scale (Wang et al. [24]). Only one clinical trial used laser-acupuncture and evaluated it as moderate methodological quality (Ebneshahidi et al. [25]). Lastly, one RCT combined acupuncture with stretching and physiotherapy techniques (microwave diathermy and myofascial release) and was evaluated as moderate quality (Georgoudis et al. [26]) (Table 3).

**Table 3:** Physiotherapy Evidence Database (PEDro) Scale

	Authors	Eligibility criteria	Random allocation	Concealed allocation	Baseline comparability	Blind subjects	Blind therapists	Blind assessors	Adequate follow-up	Intention-to-treat analysis	Between-group comparisons	Point estimates and variability	/10
1	Karst et al. (2000) [12]	NO	YES	NO	YES	YES	NO	YES	YES	NO	YES	YES	7
2	White et al. (2000) [13]	YES	YES	YES	YES	YES	NO	YES	YES	YES	YES	YES	9
3	Karst et al. (2001) [14]	NO	YES	NO	YES	YES	NO	YES	YES	NO	YES	YES	7
4	Karakurum et al. (2001) [19]	YES	YES	NO	YES	YES	NO	YES	YES	NO	YES	NO	6
5	Xue et al. (2004) [22]	YES	YES	YES	YES	YES	NO	YES	YES	NO	YES	YES	8
6	Ebneshahidi et al. (2005) [25]	YES	YES	NO	YES	YES	NO	NO	YES	NO	YES	YES	6
7	Söderberg et al. (2006) [16]	YES	YES	YES	YES	NO	NO	NO	YES	YES	YES	YES	7
8	Endres et al. (2007) [15]	YES	YES	YES	YES	NO	NO	YES	YES	YES	YES	YES	8
9	Wang et al. (2007) [24]	YES	YES	YES	YES	YES	YES	YES	YES	YES	YES	YES	10
10	Söderberg et al. (2011) [17]	YES	YES	YES	YES	NO	NO	NO	YES	YES	YES	YES	7
11	Chassot et al. (2015) [23]	YES	YES	YES	YES	YES	NO	YES	YES	YES	YES	YES	9
12	Georgoudis et al. (2018) [26]	YES	NO	NO	YES	NO	NO	NO	YES	YES	YES	YES	6
13	Gildir et al. (2019) [20]	NO	YES	NO	YES	NO	NO	YES	YES	NO	YES	YES	6
14	Kamali et al. (2019) [21]	NO	YES	NO	YES	NO	NO	NO	YES	NO	YES	YES	5
15	Schiller et al. (2021) [18]	YES	YES	YES	YES	NO	NO	NO	YES	YES	YES	YES	7

Meta-analysis

Overall, there was "high" quality evidence of these four trials, including in total 557 participants (intervention and control group) (Karst et al. [12], White et al. [13], Karst et al. [14], Endres et al. [15]). Table 4 displays the main characteristics of these studies. It is well mentioned that the average number of sessions per patient was nine, and the average duration of treatment was 5.5 weeks. The main acupuncture points were selected were GB 20, LI 4, LR3, and the optional points in the head, neck, shoulders were chosen by practitioners according to the patient's symptoms and tenderness. The small number and oldness of studies about this topic show the necessity to conduct more new and well-organized clinical and randomized controlled trials. 

**Table 4:** Characteristics for the studies included in the meta-analysis

	Study				Acupuncture		Control		Outcomes			
	Authors	Sessions in total(n)	Treatment duration (weeks)	Last Follow up	Description	Subjects (n)	Description	Subjects (n)	Headache frequency (days/month), after treatment	Headache Intensity (VAS), after treatment	Headache frequency (days/month), after last follow up	Headache Intensity (VAS), after last follow up
1	Karst et al. (2000) [12]	10 sessions	5 weeks	6 weeks after the end of the treatment	Acupuncture (GB20, LI4, LR3, GB8, GB14, GB21, GB41, UB2, UB10, UB60, LU7, TW5, ST8, ST36, ST44, DU20, Extra1)	21	Sham acupuncture	18	Acupuncutre17.5 (12.6) Sham 22.8 (10.0)	Acupuncture 4.3 (2.6) Sham 4.7 (2.4)	Acupuncture 22.1 (10.6) Placebo 22.0 (9.9)	Acupuncture 4.0 (2.5) Placebo 3.9 (2.7)
2	White et al. (2000) [13]	6 sessions	6 weeks	2 months after the end of treatment	Acupuncture (GB20, LI 4, up to four optional points in head, neck or shoulders, which had to be chosen by the practitioner according to tenderness and the patient's symptoms)	23	Sham acupuncture	23	Acupuncture 13.2 (8.4) Sham 10 (5.2)	Acupuncture 2.99 (1.49) sham 3.93 (1.62)	Acupuncture 10.4 (8) Placebo 9.2 (6.8)	Acupuncture 3 (1.44) Placebo 3.79 (1.33)
3	Karst et al. (2001) [14]	10 sessions	5 weeks	6 weeks after the end of treatment	Acupuncture ( GB20, L14, LR3 and depending on the symptoms at GB8, GB14, GB21, GB41, UB2, UB10, UB60, LU7, TW5, ST8, ST36, ST44, DU20, Extra1 )	34	Placebo needle	35	Acupuncture: 13.1 (11.3) Placebo: 16.6(11.5)	Acupuncture: 4.3(2.0) Placebo: 3.9(1.7)	Acupuncture 15.8 (11.3) Placebo 15.8 (11.1)	Acupuncture 4.0 (1.9) Placebo 4.6 (1.7)
4	Endres et al. (2007) [15]	10 sessions	6 weeks	3 months after the end of treatment	Verum points were selected from prescribed lists. Verum treatment consisted of fixed points. Needles were inserted 2–30 mm and manually stimulated to achieve De Qi	208	Sham acupuncture Needles were inserted superficially (1–3 mm) and were not stimulated, so as to avoid De Qi	195	Acupuncture 6.2 (6.7) Sham 8.5 (7.9)	-----------------	Acupunture 6.0 (6.2) Placebo 8.4 (7.9)	Acupuncture 5.35 (1.84) Placebo 5.67 (1.96)

**Figure 2:**
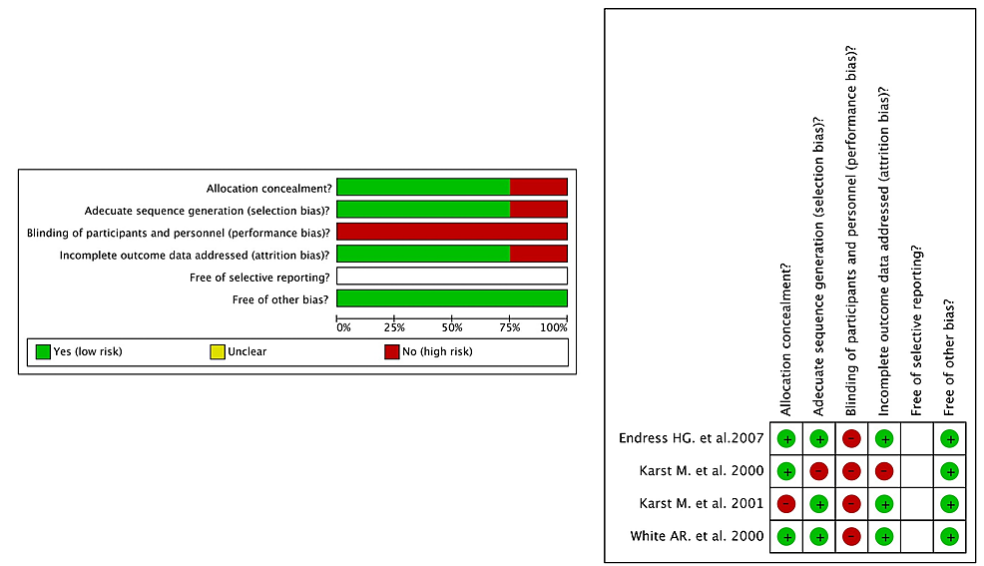
Risk-of-bias assessment of randomized controlled trials and clinical trials included in the meta-analysis

The risk of bias of the four studies included in the meta-analysis was estimated via the criteria of Grading of Recommendations Assessment, Development and Evaluation (GRADE) [10]. It was determined as low risk (Figure 2), so the true effect would probably be close to the estimated effect. For the allocation concealment, only the study of Karst et al. [14] was not in agreement with, while for the adequate sequence generation (selection bias), an earlier trial of the same author was not in compliance [12]. None of them blinded both participants and personnel (performance bias) because the techniques were hands-on, and it will be difficult for the physical therapists who provided the treatment not to distinguish the interventions performed in each group. The study of Karst et al. [12] was the only one with incomplete outcome data addressed (attrition bias). Selective reporting was not easily identified, so all of the studies were unclear about this specific criterion. Although, all of them were found free of other bias.

Frequency of headaches

**Figure 3:**
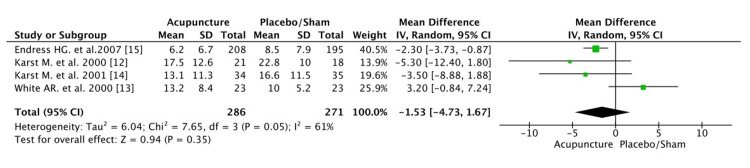
Frequency of headaches after last treatment (days/month) CI, confidence interval; df, degrees of freedom

In the meta-analysis for the frequency of headaches, all four studies were eligible. The number of days per month with a headache was evaluated after the last intervention and after the last follow-up (long term). After the last intervention, the pooled estimate for the acupuncture versus placebo/sham was not statistically significant p=0.35. This was due to the small number of participants and the small number of studies conducted. Statistical analysis suggests that headaches' frequency was not significantly lower in the acupuncture group than the placebo/sham group (mean difference -1.53 [CI: -4.73, 1.67]) (Figure 3). The heterogeneity was not high (I^2^=61%, p=0.05). Studies of Georgoudis et al. [26] support that acupuncture has beneficial effects not as monotherapy but as a part of combination therapy. Moreover, dry needling is a very effective treatment for patients with tension-type headache (Gildir et al. [20], Karakurum et al. [19]), but acupuncture, as proven in this meta-analysis, has no significant results.

**Figure 4:**
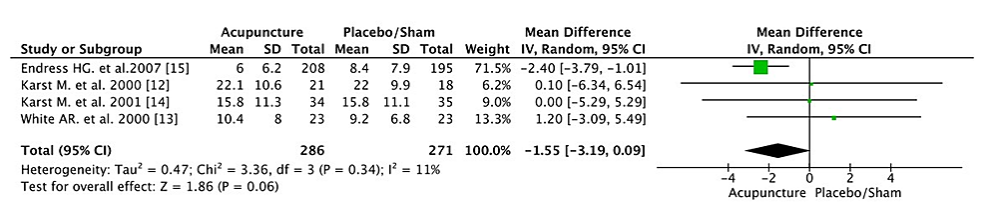
Frequency of headaches after the last follow up CI, confidence interval; df, degrees of freedom

In the long term, acupuncture seems to improve the frequency of headaches in patients with TTH, although the results were not statistically significant p=0.06. The mean last follow-up at these four studies was two months. There was a reduction of 1.55 days per month of headaches in the acupuncture group versus placebo (mean difference: -1.55 [CI: -3.19, 0.09]) (Figure 4). The heterogeneity was low (I^2^=11%, P=0.34). 

Pain intensity (VAS)

**Figure 5:**
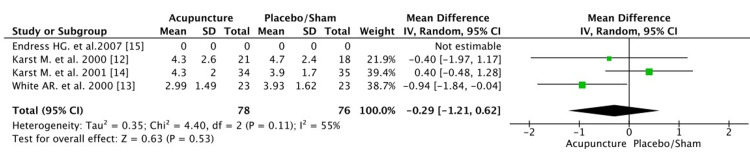
Pain intensity after last treatment CI, confidence interval; df, degrees of freedom

In the meta-analysis for the pain intensity after the last intervention, only three of the four trials were eligible. The study of Endres et al. [15] did not have sufficient data for the VAS score. The score was evaluated on a scale between 0 to 10 after the last intervention, both in the experimental and control group. Statistical analysis between the two groups suggested that the VAS score of the acupuncture group slightly reduced (-0.29) compared with the control group after the last treatment (mean difference: -0.29 [CI: -1.21, 0.62]). However, the pooled estimate between the two groups was not statistically significant p=0.53 because of the small number of studies available. The heterogeneity was not high (55%, p=0.11) (Figure 5).

**Figure 6:**
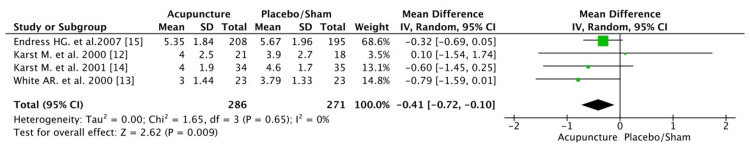
Pain intensity after the last follow-up Note: CI, confidence interval; df, degrees of freedom

In the long term, acupuncture demonstrated a statistical (p=0.009) and clinical benefit compared with placebo/sham. Statistical analyses between the two groups showed a reduction of 0.41 in the VAS scale at the acupuncture group (mean difference: -0.41[CI: -0.72, -0.10]) (Figure 6).The heterogeneity was zero (I^2^=0%, p=0.65). As a result, acupuncture seems to be an effective method for the long-term reduction of the intensity of headache pain in patients with TTH. 

## Discussion

Tension-type headaches (TTH) are the most common type of primary headaches in adults and have a significant impact on people's quality of life [1]. Though the underlying causes behind the formation of this pathology are still debatable, many theories have been formed to examine and explain TTH. Various methods have been used to treat patients with TTH, but it seems that acupuncture has become a popular intervention among them. In the 15 included studies in this review, many different acupuncture methods and techniques were described, such as acupuncture in general, sham acupuncture, laser acupuncture, electroacupuncture, and dry needling. Though only five of them examined the comparison between acupuncture and sham, and four of these were suitable for use in the meta-analysis. This research's outcomes were found to agree with the results of a previous study on the same subject conducted by Linde et al. [7] and a systematic review of systematic reviews by Huang et al. [4].

Overall, compared to sham acupuncture, acupuncture was found to have no statistically significant short-term results on the headache intensity (p=0.53) and frequency (p=0.35) in patients with TTH. An important reduction was discovered in between-group comparison after the last follow-up (1.55 days/month), but it was not significant (p=0.06). All five of the trials comparing acupuncture to placebo was characterized of high methodological quality by PEDro (Karst et al. [12], White et al. [13], Karst et al. [14], Endres et al. [15], Söderberg et al. [17]) but only four of them were used in meta-analysis (Karst et al. [12], White et al. [13], Karst et al. [14], Endres et al. [15]). The small number of the included articles in the meta-analysis and their small sample sizes could imply that heterogeneity could be high, but this is not the case as heterogeneity was not found important. Although pain intensity and headache frequency seem to decrease after the use of needles after the treatment, the results were not statistically significant. Meanwhile, after the last follow-up, both values were found to lessen, though statistically, only the pain intensity was important.

Furthermore, electroacupuncture collated to placebo was studied in three trials of high methodological quality (Xue et al. [22], Wang et al. [24], Chassot et al. [23]). Although they were found to have similar results on TTH, electroacupuncture showed a statistically significant effect on the reduction of pain intensity and headache frequency in patients with TTH except for the trial of Wang et al. [24]. Another type of acupuncture performed in one of the included researches was laser acupuncture, and the study was categorized as a moderate methodological value (Ebneshahidi et al. [25]). This intervention, when compared to placebo laser acupuncture, was found to have an important statistical effect on TTH (p<0.001). 

Four trials were included in the systematic review that compared acupuncture to various methods and techniques. Two of them that the same author conducted were categorized of high methodological quality and tested the effectiveness of acupuncture in contrast with relaxation training or physical training (Söderberg et al. 2006 [16], Söderberg et al. 2011 [17]). None of the interventions showed a statistically significant effect on TTH through relaxation training, which seems to have a higher impact on reducing headache frequency and intensity after the end of treatment compared with the other interventions. Also, the physical training group had a more significant effect at the three-month follow-up than acupuncture or relaxation training. A recent study conducted by Schiller et al. compared acupuncture to usual care or medical training and combined with medical training and was found of high methodological quality [18]. When performed additional to acupuncture and collated usual care, the results showed that medical training had a statistically significant effect (p=0.012) while both of the other treatment groups (acupuncture, medical training) differentiate significantly from usual care. Acupuncture in conjunction with stretching and physical therapy techniques (microwave diathermy and myofascial release) was found to improve further (p<0.001) TTH when compared to acupuncture and stretching without physical therapy techniques while in both groups, a statistical significance (p<0.005) was noted (Georgoudis et al. [26]).

Lastly, dry needling that was performed in three studies which were categorized of moderate methodological quality by PEDro seems to have a statistically significant effect (p<0.005) in headache frequency and intensity compared to sham dry needling (Karakurum et al. [19], Gildir et al. [20], Kamali et al. [21]). This view is in agreement with a previous systematic review conducted by France et al. [27]. In conclusion, acupuncture seems to have limited efficacy as monotherapy for reducing days of headache in patients with tension-type headache. Although, after long-term follow-up, acupuncture seems to have a statistically significant effect on pain intensity in patients with TTH. Acupuncture was found to be more efficient when combined with other treatments. Furthermore, dry needling has beneficial effects in the treatment of tension-type headaches and electroacupuncture seems to have a controversial effect on tension-type headache. Nevertheless, more studies on this topic should be conducted in order to examine thoroughly the effectiveness of acupuncture on TTH.

Limitations

This systematic review and meta-analysis certainly has some limitations. Firstly, the selection of studies was conducted in four databases with a limitation of language (English, Greek), so it is possible that some articles with the same subject could be left out. A significant number of trials were excluded because they were written in Korean, Chinese, or other Asian languages. Also, the assessment of methodological quality and evaluating the potential risk of bias of included articles was subjective as each author individually extracted the needed details. Furthermore, the number of the relevant studies found was small, and the methodological and statistical quality of a great majority was low, so consequently, the appropriate trials with complete data included in the meta-analysis were few. Another factor that should be considered is the small population of participants in most of the included studies. All of the above could respectively affect the accuracy and be the reason for the variation of the meta-analysis outcomes.

## Conclusions

Overall, after the meta-analysis of articles with high methodological quality, acupuncture's effectiveness compared to sham seems to be statistically non-significant on headache intensity and frequency in patients with TTH after the treatment. Both headache intensity and frequency were reduced in the long term, although only in the pain intensity, and the results were statistically significant. Therefore, more studies on this topic should be conducted to examine its effectiveness in headache frequency and intensity.

## References

[1] Fernández-de-Las-Peñas C, Cuadrado ML (2016). Physical therapy for headaches. Cephalalgia.

[2] Jiang W, Li Z, Wei N, Chang W, Chen W, Sui HJ (2019). Effectiveness of physical therapy on the suboccipital area of patients with tension-type headache: A meta-analysis of randomized controlled trials. Medicine (Baltimore).

[3] Pourahmadi M, Dommerholt J, Fernández-de-Las-Peñas C (2021). Dry needling for the treatment of tension-type, cervicogenic, or migraine headaches: a systematic review and meta-analysis. Phys Ther.

[4] Huang J, Shen M, Qin X, Guo W, Li H (2020). Acupuncture for the treatment of tension-type headache: an overview of systematic reviews. Evid Based Complement Alternat Med.

[5] Nielsen A (2017). Acupuncture for the prevention of tension-type headache (2016). Explore (NY).

[6] Do TP, Heldarskard GF, Kolding LT, Hvedstrup J, Schytz HW (2018). Myofascial trigger points in migraine and tension-type headache. J Headache Pain.

[7] Linde K, Allais G, Brinkhaus B (2016). Acupuncture for the prevention of tension-type headache. Cochrane Database Syst Rev.

[8] Saturni S, Bellini F, Braido F (2014). Randomized controlled trials and real life studies. Approaches and methodologies: a clinical point of view. Pulm Pharmacol Ther.

[9] Maher CG, Sherrington C, Herbert RD, Moseley AM, Elkins M (2003). Reliability of the PEDro scale for rating quality of randomized controlled trials physical therapy. Phys Ther.

[10] Guyatt G, Oxman AD, Akl EA (2011). GRADE guidelines: 1. Introduction-GRADE evidence profiles and summary of findings tables. J Clin Epidemiol.

[11] Higgins JP, Thompson SG, Deeks JJ, Altman DG (2003). Measuring inconsistency in meta-analyses. BMJ.

[12] Karst M, Rollnik JD, Fink M, Reinhard M, Piepenbrock S (2000). Pressure pain threshold and needle acupuncture in chronic tension-type headache - a double-blind placebo-controlled study. Pain.

[13] White AR, Resch KL, Chan JC, Norris CD, Modi SK, Patel JN, Ernst E (2000). Acupuncture for episodic tension-type headache: a multicentre randomized controlled trial. Cephalalgia.

[14] Karst M, Reinhard M, Thum P, Wiese B, Rollnik J, Fink M (2001). Needle acupuncture in tension-type headache: a randomized, placebo-controlled study. Cephalalgia.

[15] Endres HG, Böwing G, Diener HC (2007). Acupuncture for tension-type headache: a multicentre, sham-controlled, patient-and observer-blinded, randomised trial. J Headache Pain.

[16] Söderberg E, Carlsson J, Stener-Victorin E (2006). Chronic tension-type headache treated with acupuncture, physical training and relaxation training. Between-group differences. Cephalalgia.

[17] Söderberg EI, Carlsson JY, Stener-Victorin E, Dahlöf C (2011). Subjective well-being in patients with chronic tension-type headache: effect of acupuncture, physical training, and relaxation training. Clin J Pain.

[18] Schiller J, Karst M, Kellner T (2021). Combination of acupuncture and medical training therapy on tension type headache: results of a randomised controlled pilot study. Cephalalgia.

[19] Karakurum B, Karaalin O, Coskun Ö, Dora B, Üçler S, Inan L (2001). The ‘dry-needle technique’: intramuscular stimulation in tension-type headache. Cephalalgia.

[20] Gildir S, Tüzün EH, Eroğlu G, Eker L (2019). A randomized trial of trigger point dry needling versus sham needling for chronic tension-type headache. Medicine (Baltimore).

[21] Kamali F, Mohamadi M, Fakheri L, Mohammadnejad F (2019). Dry needling versus friction massage to treat tension type headache: a randomized clinical trial. J Bodyw Mov Ther.

[22] Xue CC, Dong L, Polus B (2004). Electroacupuncture for tension-type headache on distal acupoints only: a randomized, controlled, crossover trial. Headache.

[23] Chassot M, Dussan-Sarria JA, Sehn FC (2015). Electroacupuncture analgesia is associated with increased serum brain-derived neurotrophic factor in chronic tension-type headache: a randomized, sham controlled, crossover trial. BMC Complement Altern Med.

[24] Wang K, Svensson P, Arendt-Nielsen L (2007). Effect of acupuncture-like electrical stimulation on chronic tension-type headache: a randomized, double-blinded, placebo-controlled trial. Clin J Pain.

[25] Ebneshahidi NS, Heshmatipour M, Moghaddami A, Eghtesadi-Araghi P (2005). The effects of laser acupuncture on chronic tension headache--a randomised controlled trial. Acupunct Med.

[26] Georgoudis G, Felah B, Nikolaidis P, Damigos D (2018). The effect of myofascial release and microwave diathermy combined with acupuncture versus acupuncture therapy in tension-type headache patients: a pragmatic randomized controlled trial. Physiother Res Int.

[27] France S, Bown J, Nowosilskyj M, Mott M, Rand S, Walters J (2014). Evidence for the use of dry needling and physiotherapy in the management of cervicogenic or tension-type headache: a systematic review. Cephalalgia.

